# Aerogel‐Functionalized Thermoplastic Polyurethane as Waterproof, Breathable Freestanding Films and Coatings for Passive Daytime Radiative Cooling

**DOI:** 10.1002/advs.202201190

**Published:** 2022-04-27

**Authors:** Xiameng Shan, Ling Liu, Yusi Wu, Dengsen Yuan, Jing Wang, Chengjiao Zhang, Jin Wang

**Affiliations:** ^1^ School of Nano‐Tech and Nano‐Bionics University of Science and Technology of China Hefei 230026 P. R. China; ^2^ Key Laboratory of Multifunctional Nanomaterials and Smart Systems Suzhou Institute of Nano‐Tech and Nano‐Bionics Chinese Academy of Sciences Suzhou 215123 P. R. China; ^3^ Gusu Laboratory of Materials Science Suzhou 215123 P. R. China; ^4^ School of Textile and Clothing Nantong University Nantong 226019 P. R. China

**Keywords:** aerogel, passive radiative cooling, personal thermal management, thermoplastic polyurethane

## Abstract

Passive daytime radiative cooling (PDRC) is an emerging sustainable technology that can spontaneously radiate heat to outer space through an atmospheric transparency window to achieve self‐cooling. PDRC has attracted considerable attention and shows great potential for personal thermal management (PTM). However, PDRC polymers are limited to polyethylene, polyvinylidene fluoride, and their derivatives. In this study, a series of polymer films based on thermoplastic polyurethane (TPU) and their composite films with silica aerogels (aerogel‐functionalized TPU (AFTPU)) are prepared using a simple and scalable non‐solvent‐phase‐separation strategy. The TPU and AFTPU films are freestanding, mechanically strong, show high solar reflection up to 94%, and emit strongly in the atmospheric transparency window, thereby achieving subambient cooling of 10.0 and 7.7 °C on a hot summer day for the TPU and AFTPU film (10 wt%), respectively. The AFTPU films can be used as waterproof and moisture permeable coatings for traditional textiles, such as cotton, polyester, and nylon, and the highest temperature drop of 17.6 °C is achieved with respect to pristine nylon fabric, in which both the cooling performance and waterproof properties are highly desirable for the PTM applications. This study opens up a promising route for designing common polymers for highly efficient PDRC.

## Graphical abstract

Aerogel‐functionalized thermoplastic polyurethane freestanding films and coatings are designed and prepared by a non‐solvent‐phase‐separated strategy for efficient passive daytime radiative cooling. The films are mechanically strong and achieve a subambient cooling of 10.0 °C on a hot day. When used as coatings for traditional textiles, a radiative cooling of 17.6 °C is achieved with respect to pristine nylon fabric.

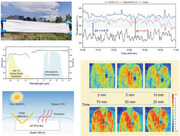

## 1 Introduction

In the last two decades, a huge quantity of energy has been consumed with the rapid development of industry and the economy, leading to an imbalance between fossil resources supply and energy demand.^[^ ^1^, ^2^, ^3^ ^]^ Over time, this has triggered a serious energy crisis and poses great pressure on the environment and natural resources.^[^ ^4^ ^]^ When space cooling is conducted in summer, a large amount of electricity is consumed because it cools the whole building rather than the human body. In addition, the use of space refrigeration equipment, such as air conditioners, wastes a large amount of energy.^[^ ^5^ ^]^ At the end of 2019, the coronavirus disease (COVID‐19) broke out and quickly spread to other parts of the world.^[^ ^6^ ^]^ People sweat profusely while queuing for nucleic acid tests during hot summers. Medical staff wore protective clothing that had poor air and moisture permeabilities.^[^ ^7^ ^]^ As the new air supply defensive clothing available in the market poses health risks, it is necessary to explore novel materials and methods to achieve cooling with low or zero energy consumption, while ensuring comfort and safety.^[^ ^8^, ^9^, ^10^ ^]^ Passive radiative cooling, a strategy without any energy consumption, is a promising approach for releasing condensed pressure for building cooling^[^ ^11^, ^12^, ^13^ ^]^ and personal thermal management (PTM).^[^ ^14^, ^15^ ^]^ The working principle of passive radiative cooling is simple; any object that radiates energy outward in the form of electromagnetic wave radiation can be cooled if the amount of heat released through radiation is greater than that received from the environment. Compared to building heating, ventilation, and air conditioning (HVAC) systems, passive radiative cooling does not require external energy to achieve high‐efficiency cooling; thus, it has attracted increasing attention in recent years. To achieve strong and effective cooling during the daytime, especially during hot summer days, the following two conditions must be met by a material:^[^ ^10^, ^11^, ^16^, ^17^ ^]^ 1) a high solar reflectance (*R*_solar_) (≈1) in the wavelength range of 0.2–2.5 µm to avoid solar absorption, which can convert to heat and increase the temperature significantly, and 2) a high emissivity (≈1) in the long‐wavelength infrared (LWIR) atmospheric transparency window (8–13 µm) for radiating heat to the cold space.

To date, various types of radiative cooling materials have been developed, including multilayered structures,^[^ ^18^, ^19^, ^20^ ^]^ metamaterials,^[^ ^21^ ^]^ randomly distributed particle structures,^[^ ^22^, ^23^, ^24^ ^]^ and porous structures.^[^ ^25^, ^26^, ^27^, ^28^, ^29^, ^30^ ^]^ The first three materials generally include high‐reflectivity metallic materials at the bottom to reflect sunlight, which are brittle and airtight; thus, they are unsuitable for PTM. Porous structures based on polymers, either in the form of textiles or films, show great potential for application in PTM. Human skin has a high IR emissivity of ≈0.98 in the range of 7–14 µm, which overlaps with the atmospheric transparency window. Therefore, materials with extremely high IR transmittance can help the heat dissipation of the human body.^[^ ^11^ ^]^ Thus, polymers with high emissivity or transmittance are promising passive daytime radiative cooling (PDRC) materials. Nevertheless, these polymers are limited to polyethylene (PE)^[^ ^31^, ^32^, ^33^, ^34^, ^35^ ^]^ for high transmittance and polyethylene oxide (PEO),^[^ ^36^ ^]^ polyvinylidene fluoride (PVDF), and its derivatives^[^ ^37^, ^38^, ^39^, ^40^ ^]^ for high emittance. Thermoplastic polyurethane (TPU) is a linear block copolymer elastomer consisting of alternating coil–rod segments, which can be processed using various techniques, such as extrusion injection, blow molding, compression molding, or solution coating. TPUs have widely been used in textiles owing to their high transparency, elasticity, tensile strength, wear resistance, and corrosion resistance.^[^ ^41^ ^]^

In this study, TPU films were designed and confirmed to be a highly efficient PDRC material using a scalable non‐solvent‐phase‐separation (NSPS) strategy. The NSPS method transformed the highly transparent TPU film into a highly reflective white film with an average *R*_solar_ of >94% and an IR emittance of >95% that could be tailored by the incorporation of superhydrophobic silica aerogels (SSA). A significant cooling performance of 10 °C and a cooling power of 40 W m^−2^ were demonstrated by the TPU film during a hot daytime. Aerogel‐functionalized TPU (AFTPU) films were also prepared by the NSPS strategy, though their cooling performances were relative lower than that of TPU; the AFTPU films possessed high contact angles and were not easily wetted by water. Therefore, AFTPU films may be more attractive for water‐resistance wearable usage for passive cooling. Apart from a self‐supporting breathable film, the film can also be used as a coating on traditional textiles, which can be easily scaled up and is waterproof and breathable. The results suggest that, by careful structural design, other types of polymers excluding PE, PEO, and PVDF may also be used for PDRC, and they may even outperform the reported PDRC polymers in both cooling performance and comfort of wearing (flexibility, waterproof, breathability, and corrosion resistance).

## 2 Results and Discussion

### 2.1 Preparation of AFTPU Films via NSPS

**Figure 1:**
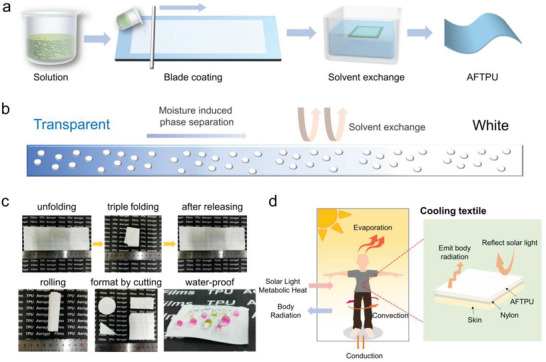
a) Fabrication process of the AFTPU films. b) Schematic of the NSPS process. c) Photographs of AFTPU‐10 film being folded and released, rolled, formatted by cutting, and with colored water drops. d) Schematic of heat input and output pathways of the human body in an outdoor environment and the working principle of the AFTPU films and coatings.

TPU films prepared using traditional methods, such as solution casting (SC), are highly transparent (Figure S1a, Supporting Information),^[^ ^42^, ^43^ ^]^ even with the incorporation of SSA up to 25 wt% (Figure S1b–d, Supporting Information). The *R*_solar_ of the TPU (SC) film was <20% (Figure S2, Supporting Information). Thus, the TPU did not exhibit any PDRC performance. To solve this problem, the NSPS method was developed in this study.^[^ ^44^, ^45^ ^]^ As shown in **Figure** **1**a,b, dimethylformamide (DMF) solutions of TPU (15 wt%) (or containing different amounts of SSA) were blade‐coated on a clean glass substrate. The films were then exposed to the air for 5 min and gradually changed from transparent to translucent. The phase‐separated films were solvent‐exchanged with water, which turned them white and opaque. Finally, the films were oven‐dried and denoted as AFTPU‐*n*, where *n* indicates the weight content of the SSA. The term “TPU film” in the following section denotes pure TPU film prepared by the NSPS method without the presence of SSA unless specified.

The AFTPU films were white, opaque, freestanding, and mechanically strong. Figure 1c shows that the AFTPU films could be folded, rolled, and completely restored to their original shapes. They could be further formatted into various shapes (circle, rectangle, triangle, etc.) simply by cutting, which may find interesting applications in wearing. Additionally, the AFTPU films were hydrophobic and could not be wetted by water. Owing to these properties, the AFTPU films may be used in PTM, as shown in Figure 1d,^[^ ^11^, ^14^ ^]^ and the body's heat input is mainly derived from the sun and metabolic heat, whereas the heat output includes conduction, convection, evaporation, and radiation. The AFTPU films and coatings used for PDRC can block the heat input from the sun and dissipate heat via radiation without any energy consumption.^[^ ^36^, ^46^ ^]^ Their properties and performances are discussed in the following sections.

### 2.2 Characterization of the AFTPU Films

**Figure 2:**
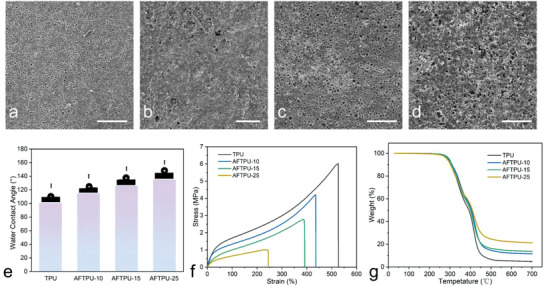
a–d) SEM images of TPU, AFTPU‐10, AFTPU‐15, and AFTPU‐25. Scale bar. 100 µm. e) Water contact angles, f) stress–strain curves, and g) TGA curves of TPU, AFTPU‐10, AFTPU‐15, and AFTPU‐25 films.

**Figure** **2**a–d shows the scanning electron microscope (SEM) images of the AFTPU films, revealing porous structures with randomly distributed disorderly microscale pores. Adjusting the SSA content did not significantly impact the porous structure, suggesting that these micropores must have resulted from the NSPS process. The energy dispersive X‐ray spectroscopy (EDS) mapping, shown in Figure S2 (Supporting Information), further suggested that the SSA were homogenously dispersed in the TPU matrix, and rough surfaces could be clearly observed from the carbon element. By contrast, the films prepared by the SC method were nonporous with smooth surfaces, even with the presence of 25 wt% SSA (Figure S3, Supporting Information). Nevertheless, the hydrophobicity of the AFTPU films increased with the increasing SSA content. As shown in Figure 2e, the contact angles of the TPU, AFTPU‐10, AFTPU‐15, and AFTPU‐25 films were 101°, 115°, 126°, and 135°, respectively. In addition, the average moisture permeabilities of the TPU, AFTPU‐10, AFTPU‐15, and AFTPU‐25 films were 861, 1014, 353, and 778 g m^−2^ for 24 h, respectively. The moisture permeabilities of the films were relatively low and were not directly related to the SSA contains, possibly due to the similar porous structures as shown in the SEM images (Figure 2a–d), which were smaller than that of traditional textiles. The results indicated that the addition of SSA not only improved the hydrophobicity but also preserved the moisture permeability, making them promising waterproof coatings for textiles.

The mechanical properties of the AFTPU films are shown in Figure 2f. The SSA‐free TPU film exhibited excellent flexibility and could be stretched by more than 500%. The increase in the SSA content of the AFTPU films resulted in decreased in mechanical strength. Nevertheless, the AFTPU‐25 film could still be stretched by 250% and behaved as a thermoplastic elastomer. The Fourier transform infrared (FT‐IR) spectra of AFTPU (Figure S4, Supporting Information) indicated that no chemical reaction occurred between SSA and TPU. Thus, SSA must be physically dispersed and embedded in the TPU matrix.^[^ ^47^, ^48^ ^]^ The soft and hard segments of TPU caused microscopic phase separation, and the hard segments acted as physical crosslinkers in the elastic chain. However, owing to the low interfacial bonding between the SSA and TPU, the mutual aggregation of particles constituted to local defects. The tensile force separated the interface between SSA and TPU, and the mechanical properties deteriorated. Thermogravimetric analysis (TGA) curves of the TPU and AFTPU films are shown in Figure 2g. The thermogram of the TPU film shows a two‐step degradation.^[^ ^42^, ^43^, ^44^, ^45^ ^]^ The degradation temperatures started at 247 and 350 °C, corresponding to the decomposition of the soft and hard segments, respectively. The residue mass was 4.8 wt%. The decomposition behaviors of the AFTPU films were almost identical to that of pure TPU; however, the residual mass significantly increased: 11.4, 13.7, and 21.4 wt% for AFTPU‐10, AFTPU‐15, and AFTPU‐25, respectively. The increase in the residual mass could be ascribed to SSA, which exhibits high thermal stability.^[^ ^49^, ^50^, ^51^ ^]^

### 2.3 Optical Properties and Outdoor Experiment of AFTPU Films

**Figure 3:**
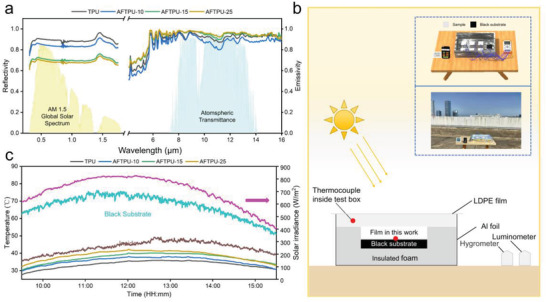
Daytime radiative cooling performance. a) Solar reflective and thermal emissive spectra of the TUP and AFTPU films. The normalized ASTM G173 global solar spectrum and atmospheric transparency window are plotted as background. b) Schematic and photograph of the setup used to evaluate the radiative cooling performance. c) Temperature tracking of the films, cavity, and black substrate. The monitored solar irradiance is included to provide primary meteorological information.

**Figure** **3**a shows the spectral reflectance and emissivity of the films according to the normalized ASTM G173 global solar spectrum and LWIR atmospheric transparency window. The average *R*_solar_ values of the TPU, AFTPU‐10, AFTPU‐15, and AFTPU‐25 films were 0.89, 0.84, 0.71, and 0.69, respectively, which were significantly higher than those of the TPU film and SSA composite TPU films (average *R*_solar_ < 0.2, the transmittance values of TPU (SC), AFTPU‐10 (SC), AFTPU‐15 (SC), and AFTPU‐25 (SC) were 0.803, 0.836, 0.843, and 0.831, respectively) prepared by the SC process (Figure S5, Supporting Information). These results confirmed that the NSPS strategy was effective in improving the reflection of the TPU film. However, the average *R*_solar_ values were reduced with the increasing SSA; the reason may be due to the fact that the SSA are highly transparent (91%),^[^ ^52^ ^]^ as shown in Figure S5 (Supporting Information), and there was no phase separation between the TPU matrix and the SSA, which means no extra interfaces that can facilitate light reflection were formed with increasing SSA. Similar tendency was also observed for the films prepared by the SC method. Additionally, the porosities of the films are calculated^[^ ^52^ ^]^ to be 69.2%, 67.6%, 58.5%, and 49.7% for TPU, AFTPU‐10, AFTPU‐15, AFTPU‐15, respectively. The solar reflectance of the TPU films increased with increasing porosity, and the similar tendency had been observed in other porous films.^[^ ^26^ ^]^ Although the increase in SSA content reduced the *R*_solar_ values, and they were relatively lower than that of PVDF,^[^ ^37^, ^38^, ^39^, ^40^ ^]^ the emissivity increased from 0.93 (TPU) to 0.96 (AFTPU‐15 and AFTPU‐25), which makes AFTPU films potential candidates for PDRC. The increase in emissivity of the AFTPU films may be due to the vibrational absorption of Si—O—Si bonds in the SSA. As illustrated by the FT‐IR spectra of the SSA (Figure S6, Supporting Information), the fingerprint area of the SSA ranged from 1300 to 600 cm^−1^, which coincides with the atmospheric transparency window (8–13 µm). The strong and highly selective emissivity of SSA may have significantly contributed to the high emissivity of the AFTPU films when the content was higher than 10 wt%.

Figure 3b shows the setup used to evaluate the PDRC performance of the films in the outdoor environment. The TPU, AFTPU‐10, AFTPU‐15, and AFTPU‐25 films were used as passive radiative coolers. The black substrate and ambient temperatures of the equipment were also measured. Five thermocouples were placed at the bottom of each sample and on the surface of the black substrate. Another thermocouple was suspended in the cavity to measure the ambient temperature. Considering the characteristics of solar radiation intensity on 18 September 2021, the time for the experiments was chosen to be from 9:30 a.m. to 3:30 p.m. The solar radiation intensity in this interval was highest during the day, and the maximum was greater than 800 W m^−2^. The PDRC results are shown in Figure 3c. The solar irradiance reached its peak at noon, and the power density exceeded 820 W m^−2^. Correspondingly, the ambient temperature and black substrate temperature increased to 45 and 75 °C, respectively. Notably, all the AFTPU films exhibited lower temperatures than the ambient temperature, and the temperature drop was directly proportional to the *R*_solar_, as discussed earlier. To clearly understand the cooling performance of the film, the average cooling temperature difference for all films (Figure S7, Supporting Information) was determined. The average temperature drops (10:50–12:50: sunlight intensity = 800 W m^−2^) of the TPU, AFTPU‐10, AFTPU‐15, and AFTPU‐25 films were 9.99, 7.68, 5.82, and 3.80 °C, respectively. Although the month was September, the weather was still hot, with the highest atmospheric temperature of 38 °C when the study was conducted. These results indicate that the TPU and AFTPU films are powerful PDRC materials, even in extremely hot weather conditions.

### 2.4 Characterization and PDRC Performance of AFTPU as Coatings

**Figure 4:**
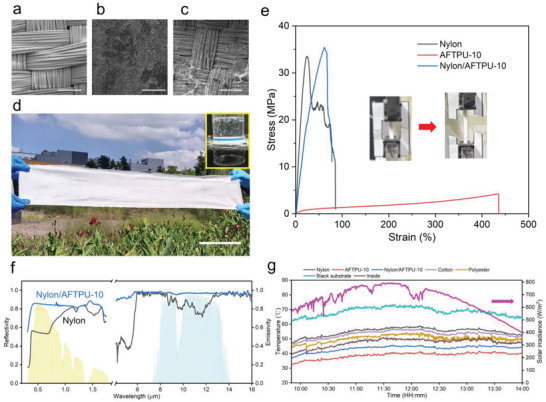
a–c) SEM images of the nylon fabric, the front site of nylon/AFTPU‐10, and the backside of nylon/AFTPU‐10. Scale bar: 200 µm. d) Photograph of the front side of nylon/AFTPU‐10. Inset: Photograph of a waterproofing and breathability test. Scale bar: 10 cm. e) Mechanical strength tests of the nylon fabric, AFTPU‐10, and the coated fabric. f) Spectral reflectivity and emissivity of nylon and nylon/AFTPU‐10. g) Temperature tracking of various fabrics, films, ambient atmosphere, and the black substrate. The monitored solar irradiance is included to provide primary meteorological information.

TPU can be used not only as a self‐supporting PDRC material but also as a coating for traditional fabrics. Considering the waterproof and moisture permeability requirement for wearing, AFTPU‐10 was used for the coating because it showed both high cooling (7.7 °C) and good water‐resistance performances. The cooling performance and mechanical property of AFTPU‐25 were relatively poor, while the waterproof property of AFTPU‐5 was limited improved as compared to that of TPU and can be wetted by water (Figure S8, Supporting Information); therefore, AFTPU‐25 and AFTPU‐5 were not used for the coating experiments in this work. The coated fabric was denoted as nylon/AFTPU‐10. As depicted in **Figure** **4**a, the nylon fabric has a distinct warp and weft structure. After coating, the surface was similar to that of AFTPU‐10 (Figure 4b), whereas the other side of the nylon fabric remained unchanged (Figure 4c; Figure S9, Supporting Information). Figure 4d shows a photograph of nylon/AFTPU‐10, which was white and could be scaled up for production. The inset image shows that the composite fabric nylon/AFTPU‐10 still had excellent breathability and waterproof function, whereas its moisture permeability was 1026 g m^−2^ for 24 h. Moreover, nylon/AFTPU‐10 had a high water contact angle of 123° (Figure S10, Supporting Information), and its FT‐IR spectrum was comparable to that of AFTPU‐10 (Figure S11, Supporting Information). The mechanical properties of the fabric also improved after the coating. The stress–strain curve, as shown in Figure 4e, indicates that the elongation at break was 25% for the nylon fabric, which increased to 60% for nylon/AFTPU‐10. The stress also improved slightly, suggesting that the tensile strength of nylon/AFTPU‐10 was higher compared to that of the AFTPU‐10 film. Because AFTPU‐10 was physically coated on nylon fabrics, the mechanical stability of nylon/AFTPU‐10 is critical for wearable use. Impressively, there were no observable changes for nylon/AFTPU‐10 after been bended 500 and 1000 times (Figure S12, Supporting Information). The SEM images of the nylon/AFTPU‐10 before and after bending for differences times also exhibited similar morphologies. The AFTPU‐10 was mainly filled in the nylon fiber pores to form an interlocked structure rather than chemically and closely coated on the nylon fibers (Figure S12, Supporting Information). On the other hand, the AFTPU‐10 was highly flexible and mainly showed the elastomer behavior similar to the TPU matrix (Figure 2f). Therefore, the nylon/AFTPU‐10 was mechanically stable and can undergo thousand times of bending without observable changes in both macro‐ and microscales.

Figure 4f and Figure S13 (Supporting Information) show the spectral properties of the nylon/AFTPU‐10 and commercial fabrics. The average *R*_solar_ of nylon/AFTPU‐10 was 0.84, which was the same as that of the AFTPU‐10 film but increased as compared to that of nylon (0.63), cotton (0.71), and polyester (0.77) fabrics. Interestingly, the average emissivity of nylon/AFTPU‐10 was 0.97, which was higher than that of the AFTPU‐10 film. This could be due to the vibration of the C—N bonds in nylon.^[^ ^17^, ^36^, ^46^ ^]^ The average emissivity of nylon was 0.86 with a lowest emissivity of 0.71 in the range of 8–13 µm, while that of the cotton and polyester fabrics were 0.91 and 0.87, respectively.

The PDRC performances of the AFTPU‐coated fabrics were evaluated on 26 September 2021, using the same setup (Figure 3b). Nylon fabric, AFTPU‐10, nylon/AFTPU‐10, cotton, and polyester fabrics were tested as potential PDRC materials, and their cooling performances were compared. Figure 4g shows the daytime temperature measurements with the accompanying solar irradiance. Under strong sunlight (12:00 pm), the temperatures of the nylon fabric, AFTPU‐10, nylon/AFTPU‐10, cotton fabric, polyester fabric, and ambient atmosphere were 57.8, 40.2, 45, 56.1, 53.5, and 49.7 °C, respectively. The temperatures of the traditional fabrics were much higher than the ambient temperature (up to 8.1 °C), and they did not exhibit any cooling effect. However, significant temperature drops were observed for AFTPU‐10 film (9.5 °C) and nylon/AFTPU‐10 (4.7 °C). Moreover, when compared to traditional fabrics, the nylon/AFTPU‐10 fabric exhibited a much lower temperature, i.e., 11.1 and 8.5 °C lower than that of cotton and polyester fabrics, respectively. The AFTPU‐10 film showed impressive temperature drops of 17.6, 15.9, and 13.3 °C as compared to nylon, cotton, and polyester fabrics, respectively. These results confirmed that the AFTPU films and coatings are promising PDRC materials for PTM on extremely hot days with sun exposure.

### 2.5 Practical Characterization of the Nylon/AFTPU‐10 with Sun Exposure

**Figure 5:**
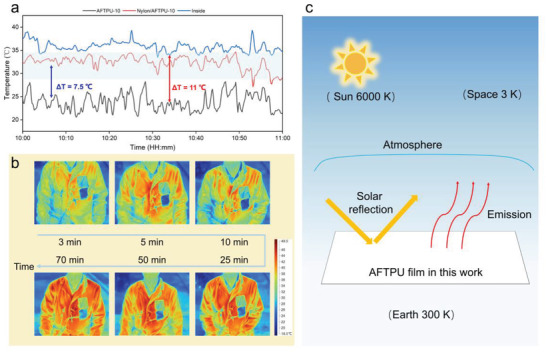
a) Temperature tracking for skin under different films in direct sunlight in Suzhou, China (31°15′N 120°43′E, 28 October 2021). The dark blue background indicates the ambient temperature. b) Infrared images of the wearing experiment under direct sunlight. c) Schematic description of the AFTPU films for daytime radiative cooling.

The application of the AFTPU films as a wearable PDRC material was demonstrated by outdoor tests. The AFTPU‐10 and nylon/AFTPU‐10 samples were sewn onto a black cotton shirt. The shirt was worn by a person who sat on a bench under direct sunlight for 70 min, and the temperature changes were monitored. With an increase in the exposure time to the sun, the surface temperature of the cloth increased. Four temperatures were measured: the ambient temperature (blue background) and the temperatures underneath AFTPU‐10, nylon/AFTPU‐10, and black shirt. It is noteworthy that the experiment was conducted on a cloudy day, and the sunlight was blocked by clouds intermittently; consequently, temperature fluctuations were observed (**Figure** **5**a). Nevertheless, the temperature of AFTPU‐10 was ≈11 °C lower than that of the cloth and 7.5 °C lower than that of nylon/AFTPU‐10. Impressively, the temperature of AFTPU‐10 was lower than the ambient temperature, confirming its radiative cooling performance on an actual wear. The lower temperature can also be clearly observed in Figure 5b, where the shirt became deep red after 70 min in sunlight (40.5 °C), whereas the color of AFTPU‐10 was still blue, corresponding to a temperature of only 22.7 °C. It is noteworthy that both the wearing experiment and outdoor setup experiment confirmed a better cooling performance of AFTPU‐10 than nylon/AFTPU‐10, possibly due to the bilayer structure of nylon/AFTPU‐10 (only one side of the nylon fabric was coated by AFTPU‐10; Figure 4b,c), whereas the bottom of nylon may slightly affect the cooling performance of the nylon/AFTPU‐10.

As shown in Figure 1d, the heat input to the outdoor environment mainly results from solar radiation.^[^ ^11^ ^]^ The AFTPU films prepared in this study possess a high *R*_solar_ and, thus, the heat gain from the sun can be significantly reduced (Figure 5c). In addition, the high emissivity of the AFTPU films and coatings overlapped with the atmospheric transparency window, through which heat could efficiently radiate to the extremely cold outer space (3 K). Thus, efficient cooling performance was demonstrated by the flexible TPU and AFTPU films.^[^ ^25^, ^27^, ^32^, ^36^ ^]^

## 3 Conclusion

Highly flexible, robust, waterproof, and breathable TPU and AFTPU films were designed and prepared via a scalable NSPS strategy. The films were white and opaque, and exhibited a high solar reflectance range (0.69–0.89) and IR emissivity (0.90–0.96). Thus, the films showed excellent PDRC performance in outdoor environments with an impressive temperature drop of ≈10 °C and a cooling power of 40 W m^−2^ under a solar radiation of 820 W m^−2^. In addition, the AFTPU film could be used as a coating for traditional textiles to achieve an impressive PDRC performance, exemplified by 4.7 °C lower than the ambient temperature, and 15.5 and 13.3 °C lower than temperatures of cotton and polyester, respectively. Compared to the reported passive radiative cooling structures, the AFTPU and TPU films reported in this study can be adapted complicated shapes by cutting, folding, trenching, etc., which make them suitable for PTM.

## 4 Experimental Section

### Materials

DMF was purchased from Sinopharm Chemical Reagent Co., Ltd. TPU (Pellethane 2363–80AE) was purchased from Lubrizol Advanced Materials, Inc. SSA were obtained from Shenzhen Yidahui Co., Ltd. The average diameter of the aerogels was 12 µm (Figure S14, Supporting Information). The specific surface area (Figure S15, Supporting Information), average pore size (Figure S16, Supporting Information), and contact angle (Figure S17, Supporting Information) of the aerogels were 1057 m^2^ g^−1^, 17 nm, and 137.5°, respectively. The anhydrous ethanol was obtained from Kunshan Chengxin Chemical Co., Ltd. All other solvents and reagents were of analytical grade and were used as received.

### Preparation of the AFTPU Films via the NSPS Method

The AFTPU and TPU films were prepared via the NSPS process as follows: taking AFTPU‐10 as an example, a TPU solution with a concentration of 15 wt% was first prepared by dissolving Pellethane 2363–80AE in DMF. Then the SSA (20 wt% with respect to the TPU) were immersed in anhydrous ethanol so that the pores of the SSA were filled with ethanol. Finally, the SSA was added to the TPU solution, followed by blade coating. A primitive TPU film was formed upon exposure to the open air for 5 min. The primitive film was then immersed in a water bath for completely phase separation. The wet AFTPU films thus obtained were solvent‐exchanged with water to replace DMF and oven‐dried at 40 °C for 18 h. The thicknesses of the AFTPU films prepared using by this method were ≈200 µm. Significant shrinkage would occur if the bladed film did not undergo NSPS for 5 min and was directly immersed in water for solvent exchange (Figure S18, Supporting Information).

### Characterizations

The morphologies of the TPU and AFTPU films were characterized by a field emission scanning electron microscope (Quanta FEG 250, FEI) with an acceleration voltage of 10 kV. The contact angles were performed using an optical angle meter system (OCA 15EC, Data Physics Instruments GmbH). The wearing comfort of the fabrics was determined by the water vapor transmission (according to GB/T 12704.2‐2009, YG601H, Ningbo Textile Instrument Factory, Zhejiang China). The tensile stress–strain curves were recorded by using an Instron 3365 tensile testing machine with a stretching rate of 10 mm min^−1^. Thermogravimetric analysis (TG 209F1 Libra, NETZSCH) was carried out to measure the decomposition temperature profile with a heating rate of 10 °C min^−1^ in a nitrogen atmosphere. The optical reflectivity of the films was measured using a UV–vis–NIR spectrophotometer (UV3600, Shimadzu Corporation). The infrared emissivity was determined using an FT‐IR spectrometer (Bruker INVENIO) with an integrating sphere (PIKE INTERRATIR). Infrared thermal images were taken with an IR camera (TiX580, Fluke). Porosities was calculated by the equation: *P* = 1 − *ρ*_film_/*ρ*_skeleton_,^[^ ^52^ ^]^ where *ρ*_film_ is the density of the TPU films and *ρ*_skeleton_ is the density of TPU polymer (1.12 g cm^−3^). The densities of the films were calculated from the volume and weight of the films.

### Cooling Performance Evaluation

The radiative cooler and various reference fabrics were tested on the roof of a five‐storied building to ensure full access to the open sky and to exclude the thermal radiation from surrounding buildings. The experimental setup was prepared according to the literature, and mainly consisted of a polystyrene foam box, aluminum foil, low‐density polyethylene (LDPE) film, radiative cooler, high‐temperature polyimide tape, thermocouples, and a luminometer.^[^ ^40^, ^53^, ^54^, ^55^ ^]^ During the outdoor experiments, the relative humidity was 40–80%, and the setup was studied under sunlight on sunny and noncloudy days. As shown in Figure 3b, to reduce the temperature of other areas of the foam box owing to heat absorption, the foam box was wrapped with aluminum foil. A piece of transparent 0.013 mm thick LDPE film was applied on top of the thermal isolation box to reduce heat convection and conduction between the cavity and the environment. The size of the cavity of the isolation box was 44 × 38 × 4.5 cm (length × width × height). Each sample measured 60 × 60 mm. Furthermore, 0.063 mm thick tape was used to cover the edges of the sample and prevent heat loss. Temperatures were measured using thermocouples placed between the films and the black substrate (Figure 3b), and the thermocouples were closely contacted with the film by an adhesive tape, whereas the temperatures of the chamber and black substrate were monitored by the thermocouple suspended in the cavity and that placed on the surface of the black substrate, respectively. Temperature data were stored every 10 s in a USB flash drive using a handheld multichannel thermometer (JK808). Simultaneously, the solar irradiance was recorded by a solar power meter (TES‐1333).

### Cooling Power Calculations

To calculate the cooling power of the films, COMSOL was used to perform heat transfer. When the films in this study were exposed to a cloudless and clear sky, they could reflect most of the sunlight. Simultaneously, due to the temperature differences between the radiative cooler and the surrounding environment, there will be heat exchange between the environment and the cooling material through convection and conduction.^[^ ^18^, ^28^, ^56^, ^57^ ^]^

Here, the radiative cooling power, *P*_cool_, is defined as

(1) $$P_{\mathrm{cool}}\left(T\right)\;=\;P_{\mathrm{rad}}\left(T\right)\;-\;P_{{}_{\mathrm{atm}}}\left(T\right)\;-\;P_{\mathrm{Sun}}\;-\;P_{\mathrm{cond}+\mathrm{conv}}$$

In Equation (1), the power radiated by the structure is given by

(2) $$P_{\mathrm{rad}}\left(T\right)\;=\;A\int d\Omega \cos\theta \int_{0}^{\infty }d\lambda I_{\mathrm{BB}}\left(T,\lambda \right)\varepsilon \left(\lambda ,\theta \right)$$

In Equation (2), $\int d\Omega \;=\;2\pi \int_{0}^{\pi /2}d\theta \sin\theta$is the angular integral over a hemisphere. $I_{\mathrm{BB}}\left(T,\lambda \right)\;=\;\frac{2hc^{2}}{\lambda^{5}}\frac{1}{e^{hc/(\lambda k_{B}T)}\;-\;1}$ is the spectral distribution of the thermal energy radiated by a blackbody at any temperature *T*, where *h* is the Planck's constant, *k*_B_ is the Boltzmann constant, *c* is the speed of light, and *λ* is the wavelength, *ɛ*(*λ*,*θ*) is the spectral and angular emissivity of the radiative cooler of surface area *A* at any temperature *T*

(3) $$P_{\mathrm{atm}}\left(T_{\mathrm{amb}}\right)\;=\;A\int d\Omega \cos\theta \int_{0}^{\infty }d\lambda I_{\mathrm{BB}}\left(T_{\mathrm{amb}},\lambda \right)\varepsilon \left(\lambda ,\theta \right)\varepsilon_{\mathrm{amb}}\left(\lambda ,\theta \right)$$

Equation (3) means absorbed power due to incident atmospheric thermal radiation.

*P*_sun_ is the absorbed power by the films from incoming solar irradiance, which is defined as

(4) $$P_{\mathrm{Sun}}=A\int_{0}^{\infty }d\lambda \varepsilon \left(\lambda ,\theta_{\mathrm{Sun}}\right)I_{\mathrm{AM}1.5}\left(\lambda \right)$$

In the above two formulas, according to Kirchhoff's law of thermal radiation, under the condition of thermodynamic equilibrium, a material's absorptivity is equal to its emissivity. And the atmospheric emissivity is given by *ε*_atm_(*λ*,*θ*) = 1 − *t*(*λ*)1/cos*θ*, where *t*(*λ*) is the atmospheric transmittance in the zenith direction. In Equation (4), *I*_AM1.5_(*λ*) is the standard solar irradiance; the structure is assumed to face the sun at a fixed angle *θ*_Sun_, Thus, the term *P*_Sun_ does not have an angular integral, and the structure's emissivity is represented by its value at *θ*_Sun_

(5) $$P_{\mathrm{cond}+\mathrm{conv}}\left(T,T_{\mathrm{amb}}\right)=Ah_{c}\left(T_{\mathrm{amb}}-T\right)$$

In Equation (5), *h*_c_ is a coefficient of the nonradiative, which combined conduction and convection.

## Conflict of Interest

The authors declare no conflict of interest.

## Supporting information

Supporting Information

## Data Availability

The data that support the findings of this study are available in the supplementary material of this article.
