# Supplementary material for: Aerogel‐Functionalized Thermoplastic Polyurethane as Waterproof, Breathable Freestanding Films and Coatings for Passive Daytime Radiative Cooling
Source: Adv Sci (Weinh). 2022 Apr 27;9(20):2201190. doi: 10.1002/advs.202201190 (PMC9284144; doi:10.1002/advs.202201190)
Supplement: Supplementary file 1 — Supporting Information [file ADVS-9-2201190-s001.pdf]

ADVANCED SCIENCE
Open Access

Supporting Information

for *Adv. Sci.*, DOI 10.1002/advs.202201190

Aerogel-Functionalized Thermoplastic Polyurethane as Waterproof, Breathable Freestanding Films and Coatings for Passive Daytime Radiative Cooling

*Xiameng Shan, Ling Liu, Yusi Wu, Dengsen Yuan, Jing Wang, Chengjiao Zhang and Jin Wang**

Supporting Information

Aerogel functionalized thermoplastic polyurethane as waterproof, breathable free-standing films and coatings for passive daytime radiative cooling

*Xiameng Shan, Ling Liu, Yusi Wu, Dengsen Yuan, Jing Wang, Chengjiao Zhang, and Jin Wang**

X. Shan, L. Liu, J. Wang, Prof. J. Wang

School of Nano-Tech and Nano-Bionics, University of Science and Technology of China, Hefei 230026, P. R. China

X. Shan, L. Liu, Y. Wu, J. Wang, Prof. J. Wang

Key Laboratory of Multifunctional Nanomaterials and Smart Systems, Suzhou Institute of Nano-Tech and Nano-Bionics, Chinese Academy of Sciences, Suzhou 215123, P. R. China

E-mail: jwang2014@sinano.ac.cn

D. Yuan

Gusu Laboratory of Materials Science, Suzhou 215123, P. R. China

Prof. C. Zhang

School of Textile and Clothing, Nantong University, Nantong 226019, P. R. China

Experimental

Fabrication of transparent TPU film *via* the solution casting method:

The preparation of the transparent TPU films can be divided into three steps: the preparation of SiO_2 aerogel dispersions, the preparation of SiO_2-TPU mixed solutions, and the preparation of TPU films by solution casting method. Taking AFTPU-10 (SC) as an example: A TPU solution with a concentration of 15 wt.% was first prepared by dissolving Pellethane 2363-80AE in DMF. Then the SSA (10 wt.% with respect to the TPU) was immersed in anhydrous ethanol so that the pores of SSA were filled with ethanol. The SSA were then mixed with the TPU solution and filled in a PTFE mold. Finally, AFTPU-10 (SC) was obtained after oven-dried at 80 °C for 18 h. The films are named TPU (SC), AFPU-10 (SC), AFPU-15 (SC), and AFPU-25 (SC) corresponding to the mass content of SSA of 0, 10, 15, and 25 wt.%, respectively.

Supplementary Figures:

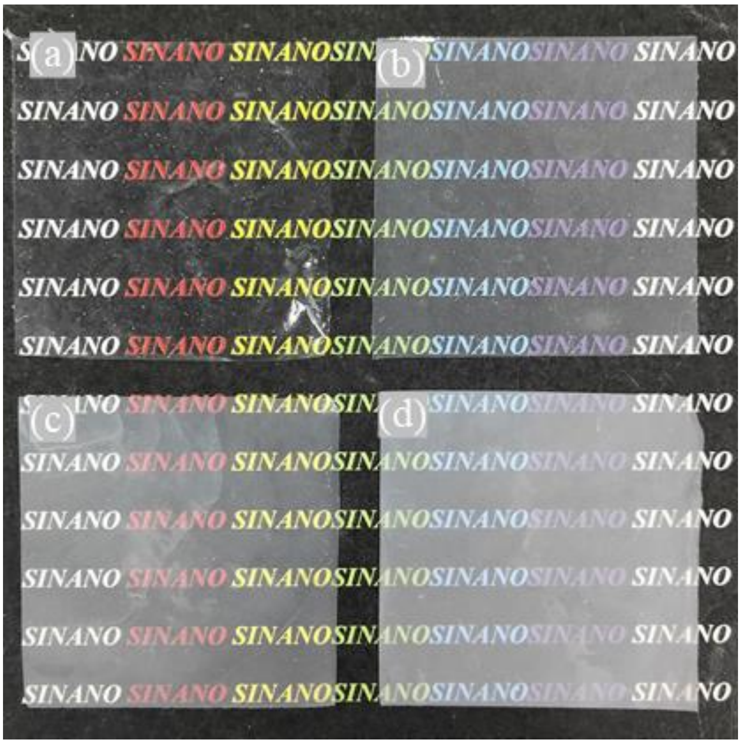

Figure S1. Photo images of the TPU films prepared by solution casting: (a) TPU (SC); (b) AFTPU-10 (SC); (c) AFTPU-15 (SC); (d) AFTPU-25 (SC).

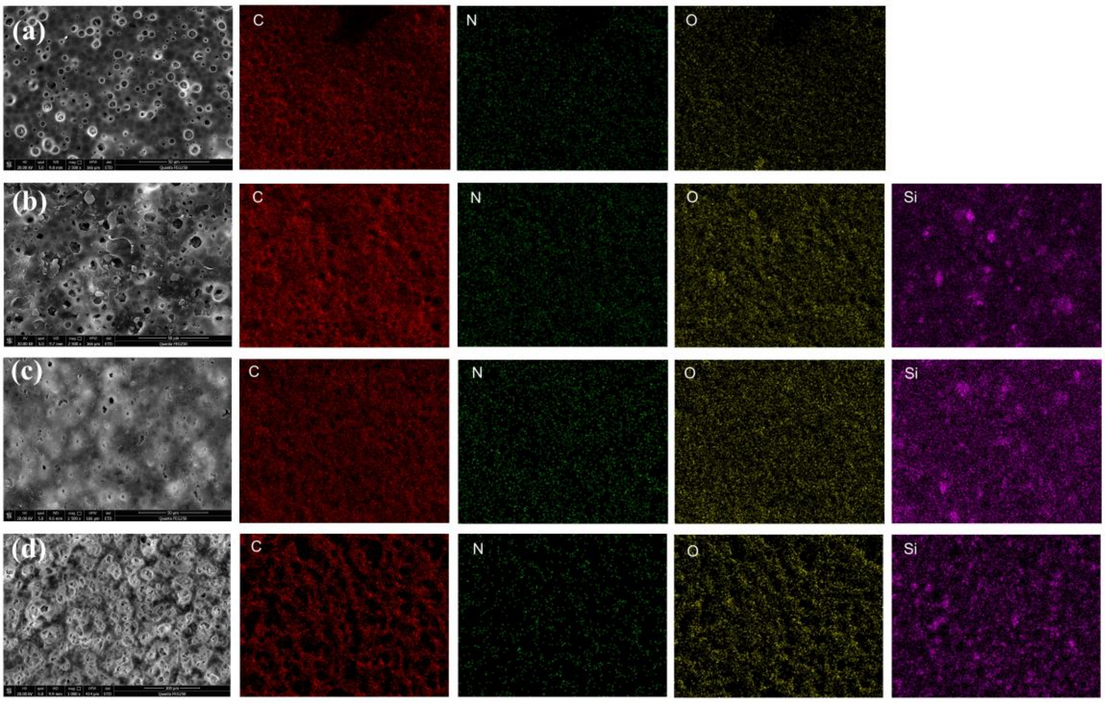

Figure S2. SEM and its corresponding EDS mapping of the films: (a) TPU, (b) AFTPU-10, (c) AFTPU-20, and (d) AFTPU-25.

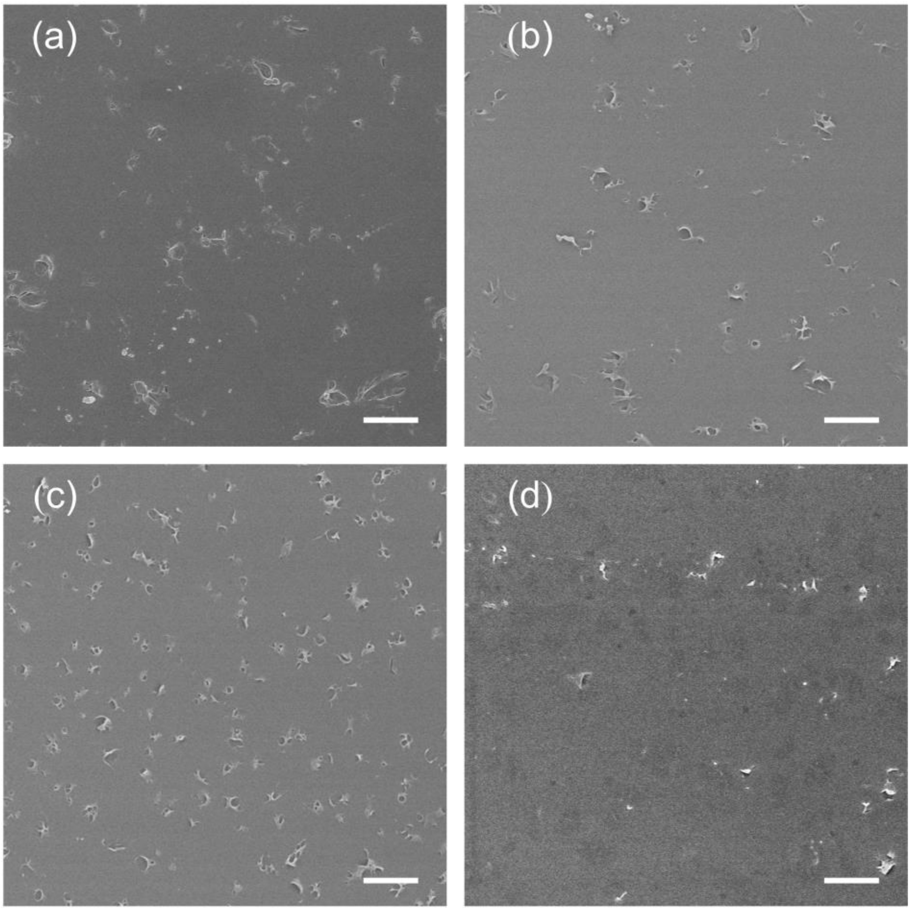

Figure S3. SEM images of TPU (SC), AFTPU-10 (SC), AFTPU-15 (SC), and AFTPU-25 (SC), the scale bar is 10 μm.

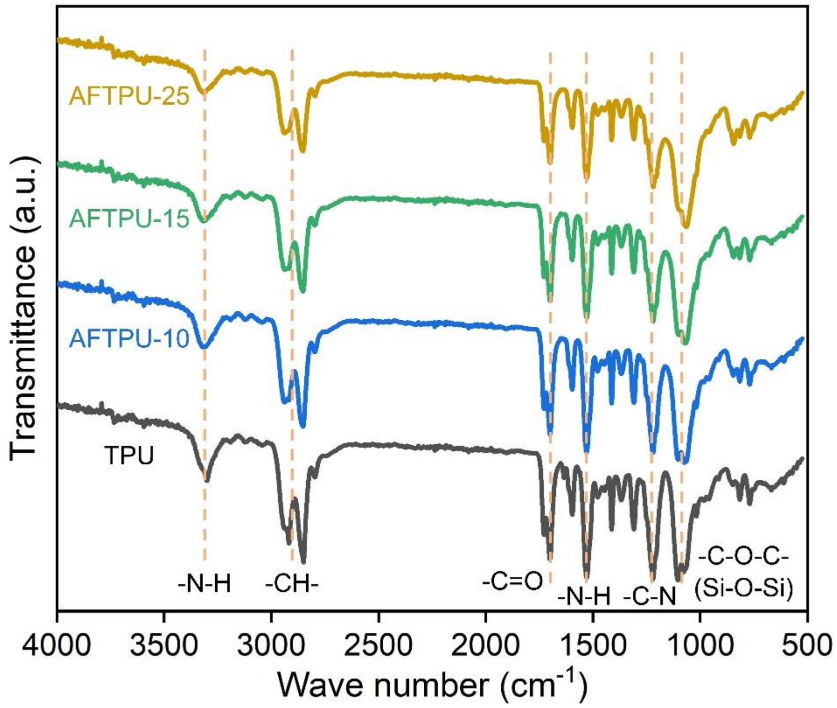

Figure S4. FT-IR spectra of TPU, AFTPU-10, AFTPU-15, and AFTPU-25 films.

As shown in Figure S4, the stretching vibration absorption peak of -N-H appears near 3310 cm^{-1} indicating that -N-H on the thermoplastic polyurethane elastomer has formed a hydrogen bond, 2900 cm^{-1} is the asymmetric stretching absorption peak of -CH. In addition, the infrared absorption peak near 1710 cm^{-1} is the characteristic absorption of polyurethane -C=O, which belongs to the stretching vibration of free carboxyl groups. 1530 cm^{-1} belongs to the -N-H deformation vibration absorption peak, and 1220 cm^{-1} corresponds to the vibration absorption peak of -C-N. Above all, the sharper peak at 1100 cm^{-1} is the -C-O-C- vibration peak of polyether polyurethane, after adding silica aerogel, because the vibration peak of Si-O-Si of aerogel molecule is also around 1100 cm^{-1}, the peak intensity at this place increases.

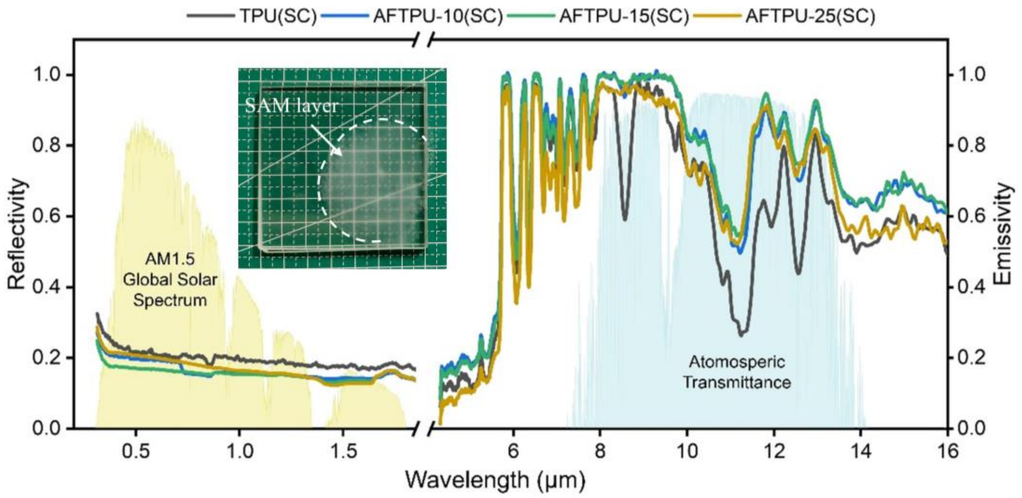

Figure S5. Spectral reflectivity and emissivity of TPU films made by solution casting with a varying weight fraction (0 - 25 wt%) of silica aerogel. Inset shows the photo image of the SAM layer sandwiched between glass slides.

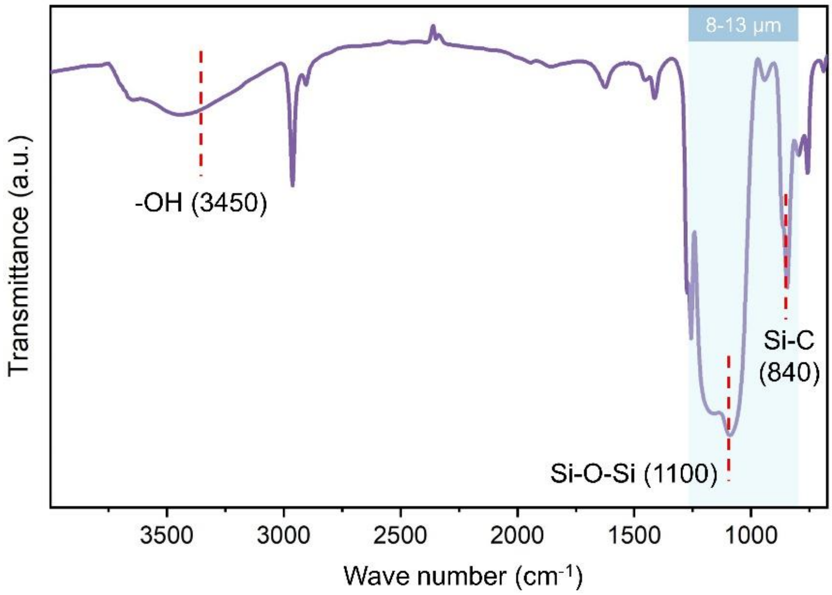

Figure S6. FT-IR spectrum of the superhydrophobic silica aerogel been used in this work.

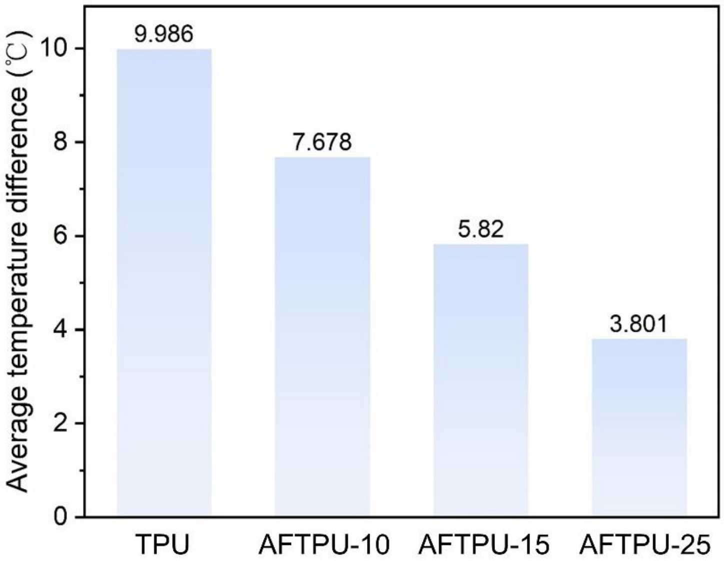

Figure S7. The average value of the temperature drops during the daytime of the samples compared with the inside air circumstance.

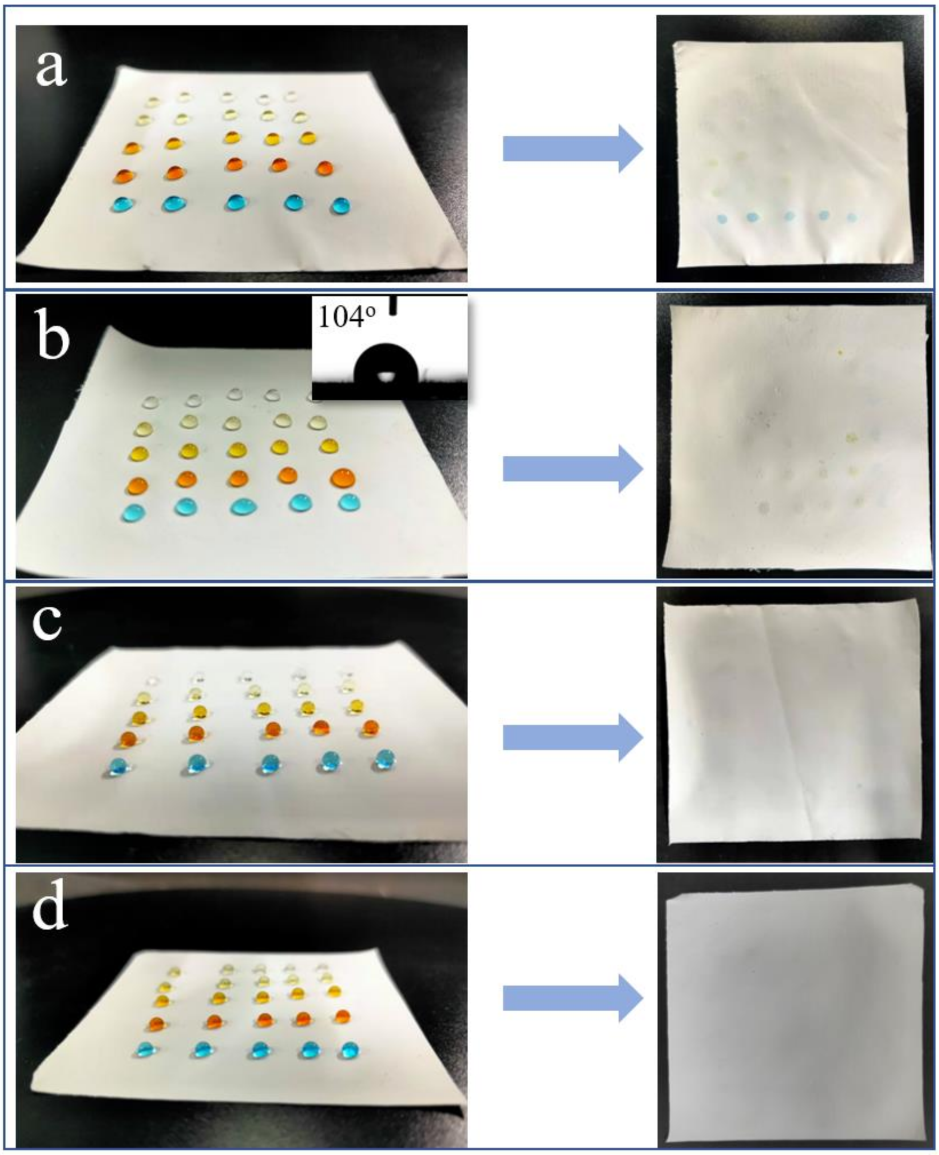

Figure S8. Wetting experiments of the films: (a) TPU, (b) AFTPU-5 (inset is the contact angle of AFTPU-5), (c) AFTPU-10, and (d) AFTPU-25.

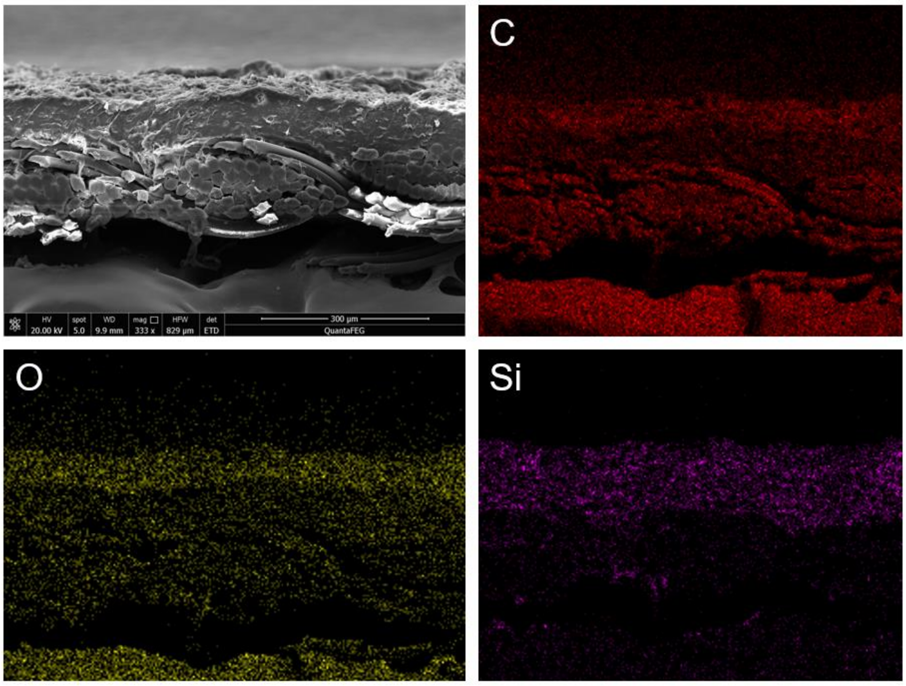

Figure S9. EDS mapping of nylon/AFTPU-10 (side view).

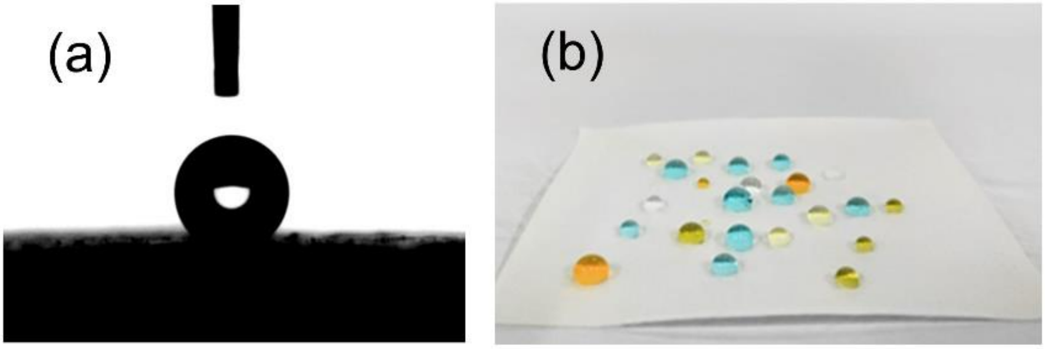

Figure S10. (a)Water contact angle of nylon/AFTPU-10; (b) Water-proof schematic of nylon/AFTPU-10.

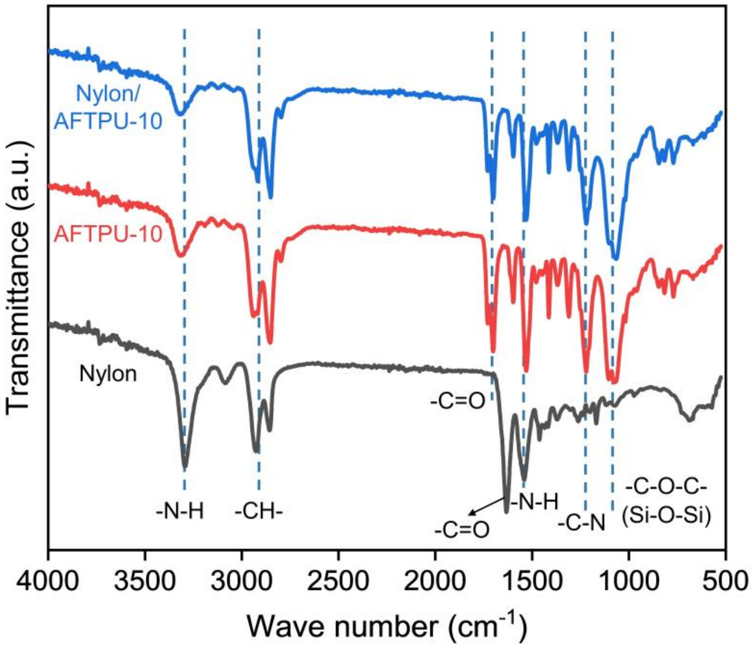

Figure S11. FT-IR spectra of nylon, AFTPU-10, nylon/AFTPU-10 films.

In Figure S11, nylon has neither the -C-O-C vibrational peak nor the sharp Si-O-Si vibrational peak at 1100 cm^{-1}, and the infrared vibrational absorption peaks of nylon/AFPU-10 and AFPU-10 are maintained consistently, which demonstrated that AFPU-10 was successfully attached to the nylon surface.

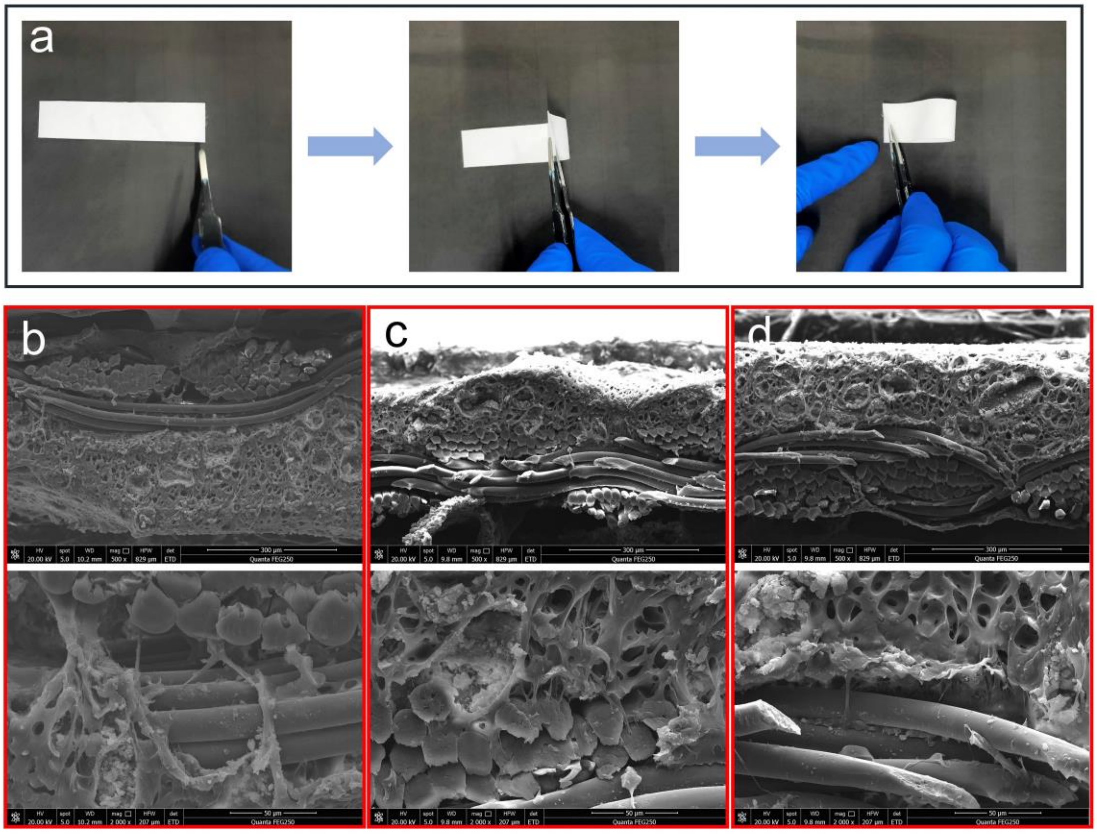

Figure S12. (a) Photo images showing the bending process of the nylon/AFTPU-10 film, the films had been bended for 180°. (b) SEM images of the pristine nylon/AFTPU-10 film. (c) SEM images of the nylon/AFTPU-10 film after bended for 500 times. (d) SEM images of the nylon/AFTPU-10 film after bended for 1000 times

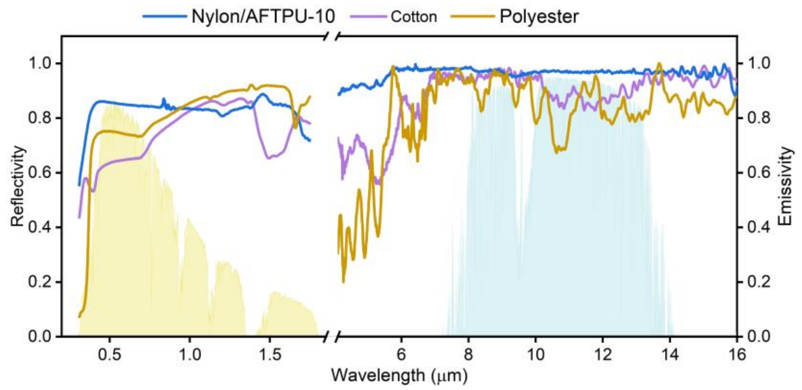

Figure S13. Spectral reflectivity and emissivity of nylon/AFTPU-10, cotton, and polyester.

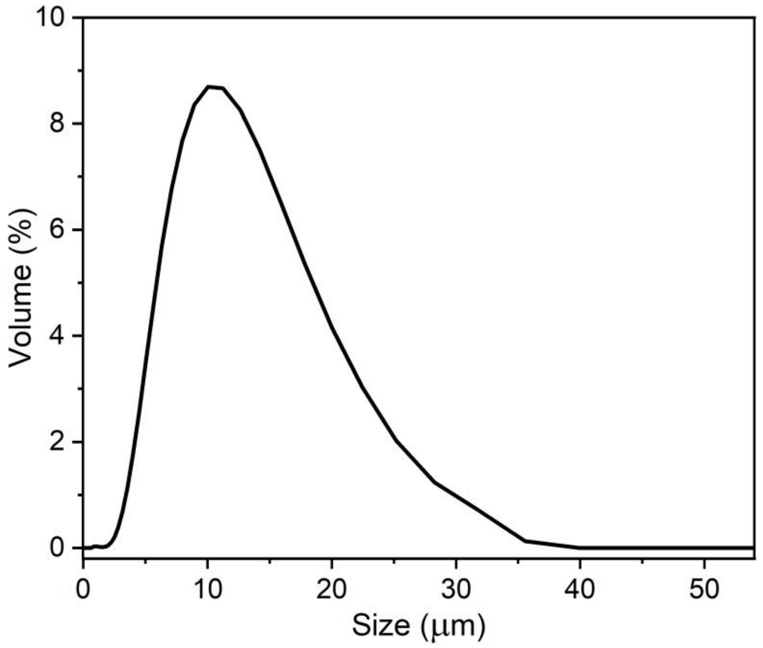

Figure S14. Particle size distribution of silica aerogel.

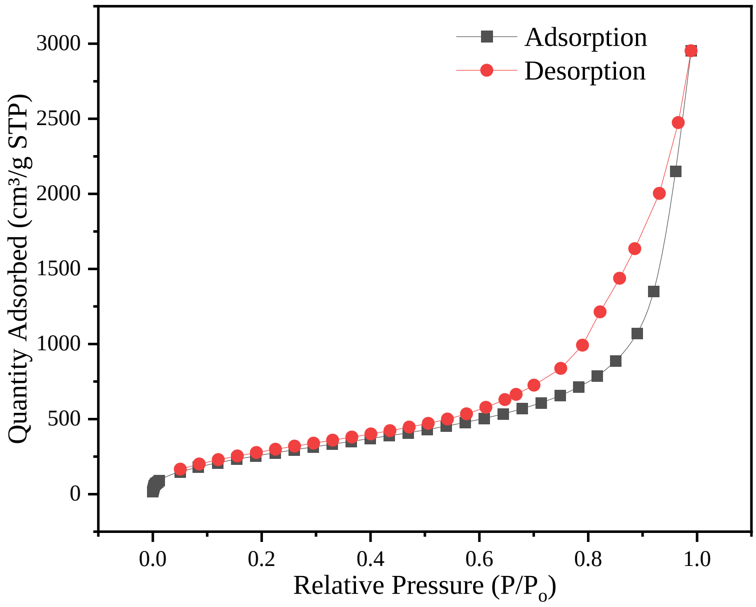

Figure S15. N_2 adsorption-desorption isotherm of the silica aerogels.

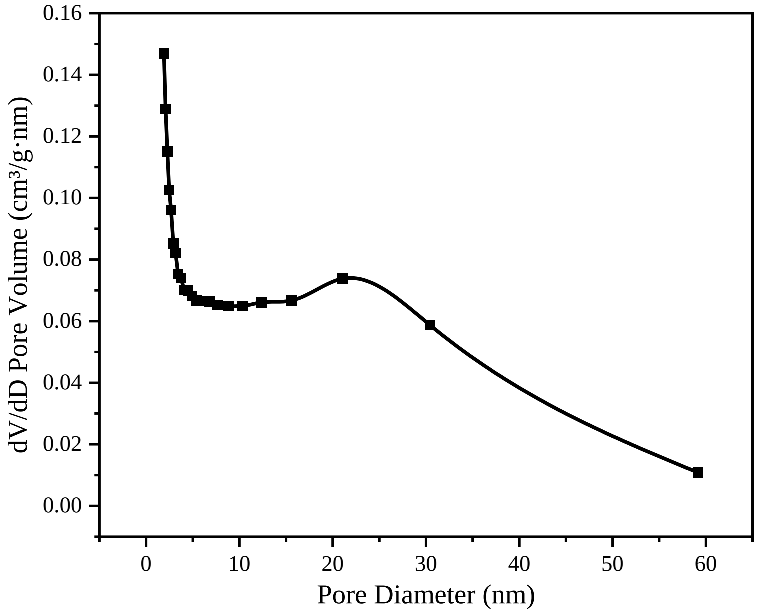

Figure S16. The pore diameter distribution of the silica aerogels.

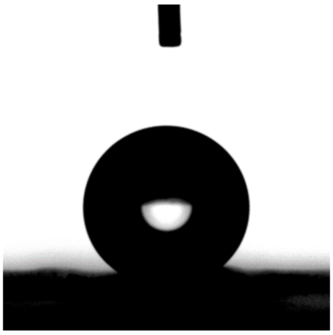

Figure S17. The water contact angle of the silica aerogels.

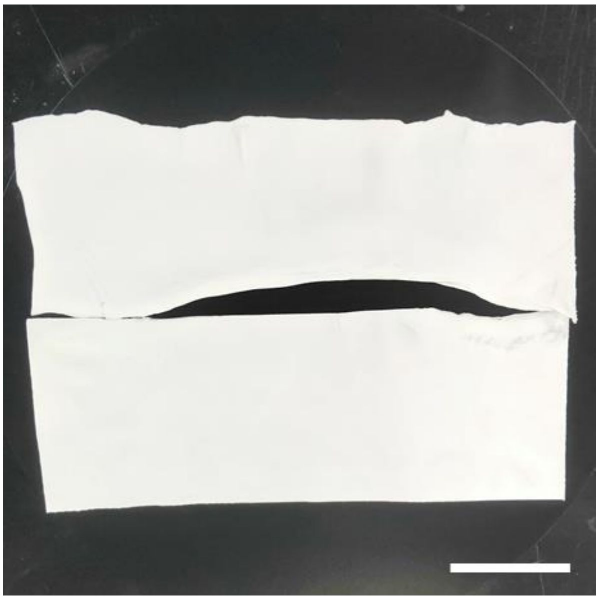

Figure S18. Photo images of TPU film prepared by the NSPS method (bottom) and by direct solvent-exchange (up). Scale bar. 5 cm.
